# PPA TELE-SAVVY: RESULTS OF AN ONLINE PILOT INTERVENTION WITH CAREGIVERS OF PERSONS WITH PRIMARY PROGRESSIVE APHASIA

**DOI:** 10.1093/geroni/igad104.2840

**Published:** 2023-12-21

**Authors:** Darby Morhardt, Kathryn Maley, Lauren Dowden, Kaitlyn O’Neil, Deborah Dyslin-Kman, Angela Roberts, Allison Lindauer, Tamar Gefen

**Affiliations:** Northwestern University Feinberg School of Medicine, Chicago, Illinois, United States; Northwestern University Feinberg School of Medicine, Chicago, Illinois, United States; Northwestern University Feinberg School of Medicine, Chicago, Illinois, United States; Northwestern University Feinberg School of Medicine, Chicago, Illinois, United States; Northwestern Medicine, Chicago, Illinois, United States; University of Western Ontario, London, Ontario, Canada; Oregon Health & Science University, Portland, Oregon, United States; Northwestern University Feinberg School of Medicine, Chicago, Illinois, United States

## Abstract

Primary Progressive Aphasia (PPA) results from a neurodegenerative disease such as frontotemporal lobar degeneration or Alzheimer’s disease and is characterized by a progressive loss of specific language functions eventually progressing to widespread cognitive decline consistent with generalized dementia. Most interventions available to dementia caregivers do not match PPA caregiving families’ needs for tailored psychosocial support. This pilot study is an adaptation of Tele-Savvy, an evidence-based online psychoeducation caregiver program. Goals: (1) address communication and other cognitive and behavioral challenges facing informal caregivers of those living with PPA throughout disease progression, and (2) help caregivers achieve competence in their role. Nine spousal caregivers were recruited to a pilot mixed methods study, which included seven 90-minute weekly videoconference sessions, mindfulness exercises and homework assignments. Participants provided feedback throughout the intervention. Pre-post effects were assessed on PPA knowledge, mood, caregiver burden, perceived stress, competence, quality of life and dyadic relationship. A focus group was held 4 weeks post-intervention. Pre-post results revealed a post-intervention trend toward decreased depression and reported relationship strain. Thematic analysis of recorded videos and transcribed weekly sessions, in addition to an evaluation focus group revealed four themes: a) Increasing knowledge of PPA; b) Learning communication and connection strategies; c) Initiating care planning; d) Integrating intellectual and emotional aspects of PPA caregiving. Participants were provided findings for their feedback. This study demonstrated the feasibility of offering an adaptation of the Tele-Savvy intervention for caregivers of persons with PPA. An informed second pilot study will be held in Spring 2023.

